# A Rehabilitation of Pixel-Based Spectral Reconstruction from RGB Images

**DOI:** 10.3390/s23084155

**Published:** 2023-04-21

**Authors:** Yi-Tun Lin, Graham D. Finlayson

**Affiliations:** School of Computing Sciences, University of East Anglia, Norwich NR4 7TJ, UK

**Keywords:** spectral reconstruction, hyperspectral imaging, multispectral imaging, inverse problem

## Abstract

Recently, many deep neural networks (DNN) have been proposed to solve the spectral reconstruction (SR) problem: recovering spectra from RGB measurements. Most DNNs seek to learn the relationship between an RGB viewed in a given spatial context and its corresponding spectra. Significantly, it is argued that the same RGB can map to different spectra depending on the context with respect to which it is seen and, more generally, that accounting for spatial context leads to improved SR. However, as it stands, DNN performance is only slightly better than the much simpler pixel-based methods where spatial context is not used. In this paper, we present a new pixel-based algorithm called A++ (an extension of the A+ sparse coding algorithm). In A+, RGBs are clustered, and within each cluster, a designated linear SR map is trained to recover spectra. In A++, we cluster the spectra instead in an attempt to ensure neighboring spectra (i.e., spectra in the same cluster) are recovered by the same SR map. A polynomial regression framework is developed to estimate the spectral neighborhoods given only the RGB values in testing, which in turn determines which mapping should be used to map each testing RGB to its reconstructed spectrum. Compared to the leading DNNs, not only does A++ deliver the best results, it is parameterized by orders of magnitude fewer parameters and has a significantly faster implementation. Moreover, in contradistinction to some DNN methods, A++ uses pixel-based processing, which is robust to image manipulations that alter the spatial context (e.g., blurring and rotations). Our demonstration on the scene relighting application also shows that, while SR methods, in general, provide more accurate relighting results compared to the traditional diagonal matrix correction, A++ provides superior color accuracy and robustness compared to the top DNN methods.

## 1. Introduction

**Figure 1:**
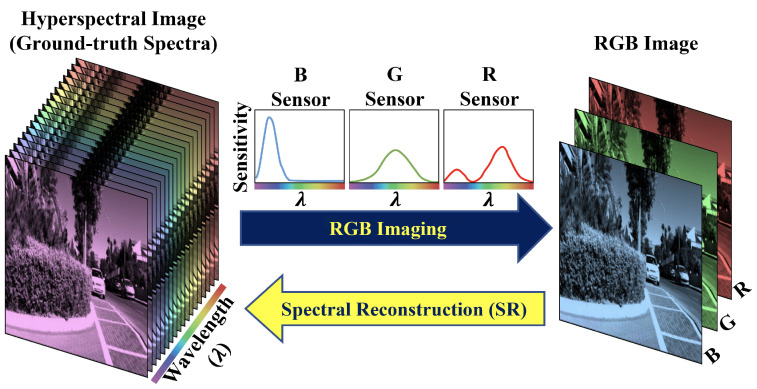
The RGB sensing coarsely sums the spectral intensities into 3 values per pixel. Conversely, spectral reconstruction recovers the lost spectral information from the RGB image.

Almost all consumer RGB cameras record 3 intensity values per pixel. These cameras use three types of color sensors with different weighting functions (called spectral sensitivity functions or camera response functions) that weighted-sum the incoming spectral signals over roughly the red, green and blue spectral regions (Figure 1 upper arrow). However, compared with RGBs, the spectrum (from which the RGB is formed [1]) conveys significantly more information about an object’s material properties. Consequently, in many computer vision tasks, it is useful to deploy *hyperspectral cameras* where finely-sampled light spectrum is captured at every pixel of the scene, including remote sensing [2,3,4,5], anomaly detection [6,7,8,9], medical imaging [10,11], food processing [12,13,14] and artwork preservation [15,16].

Despite the wide usage, traditional hyperspectral techniques [17,18] (where spectra are physically and accurately measured) are often expensive, not mobile (difficult to deploy outside the lab), and subject to low light sensitivity, low spatial resolution and/or long integration time. Many recent hyperspectral camera models resort to compressive imaging solutions [19,20,21,22,23,24,25], where the spectral information is encoded spatially as part of the captured 2D image, and some “decompressing” algorithms are used to restore the hyperspectral information. These designs realize lower-cost, higher-speed and more compact hyperspectral imaging, but nevertheless, they still require specialized physical optics, which limits their usefulness on the already widespread devices, for example, mobile phones and digital cameras. Instead of creating a new device, in spectral reconstruction (SR), we recover hyperspectral signals directly from the RGB camera responses (Figure 1 lower arrow).

Historically, SR was limited to training a “pixel-based” mapping where the RGB at each pixel is mapped to its spectral estimate independent of other pixels [26,27,28], whereas recently deep neural networks (DNN) adopt “patch-based” mappings, where image content information is (expected to be) extracted from large image patches and utilized as a part of the SR process [29,30].

On the surface, it seems the DNNs have rather a strong advantage over the legacy pixel-based methods since DNNs are built with much more powerful processing and mapping architectures, and their input information increases from pixel-RGB to an extended patch region of an RGB image. Moreover, it is sometimes argued that somewhere deep in the DNN mapping, the network can recognize materials and objects, and it is this recognition process that helps recover spectra. Tantalizingly, because of the link to the spatial context, it is sometimes claimed that DNNs can map the same RGB viewed in a different context to different spectra, solving the metamerism problem [31].

Yet, research shows that a simple pixel-based “polynomial regression” provides an SR accuracy that is only roughly 10% worse than a top DNN method [32]. This being said, it seems the idea that large image patches really bring in much useful information to SR should be challenged. Indeed, if incorporating local context into SR was found not to be helpful, then it should suffice if we revert to using the much simpler pixel-based methods (which have fewer model parameters, can be trained on the smaller data set, and run in less time compared with the current best DNN approaches). This is especially true if this 10% gap can be further lessened or indeed if the pixel-based approach can be shown to deliver better performance than DNNs.

**Figure 2:**
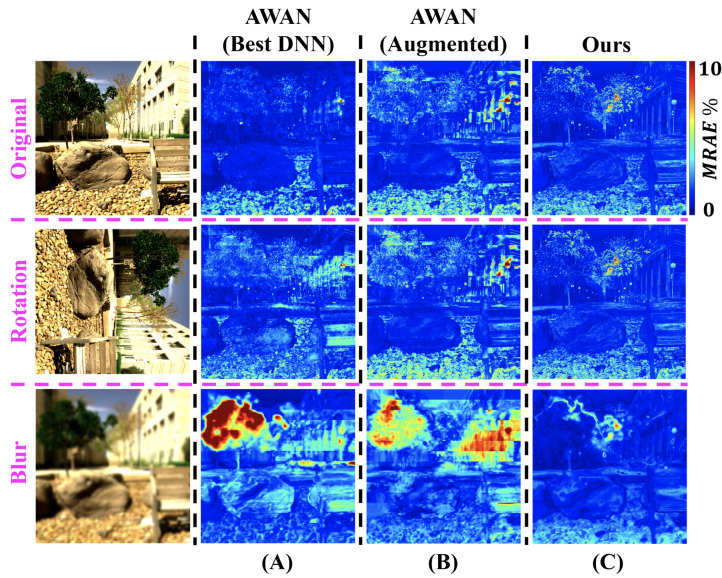
The SR mean-relative-absolute error (MRAE) maps of (**A**) the leading deep neural network (DNN) “AWAN” [35], (**B**) our data-augmented AWAN and (**C**) our pixel-based “A++”, under the original, rotation and blur conditions. The error maps of the “rotation” experiments are rotated back to upright orientation to ease comparison.

Another way to challenge existing DNNs is to look at their robustness. As most DNNs learn from image patches, it is easier for them to overfit to well-captured image contents (compared to the pixel-based SRs where image contents are not involved). For example, the contemporary DNN-based SRs do not work as well when the exposure of the image changes [33,34]. See column (A) of Figure 2, where in this paper we tested the best DNN [35] (i.e., the winner of NTIRE 2020 Spectral Reconstruction Challenge [30]) with rotated or blurred input images, and discovered that its performance considerably degraded.

Although generally, a DNN’s lack of robustness can be mitigated via data augmentation, the increase in data complexity might negatively affect the DNN’s overall performance (e.g., this is true for maintaining the exposure invariance of the DNNs [36]). Given that it is already such a close race between pixel-based and DNN-based SRs, we must re-compare both approaches—after data-augmenting the best DNN, and under the desired realistic imaging conditions.

In this paper, we challenge ourselves to achieve state-of-the-art SR performance without the help of DNN and patch-based mapping. We extend from a sparse coding method, A+ [37], where localized SR mappings are applied in different RGB neighborhoods. Our method, called A++, uses a polynomial regression SR [32] to map all RGBs to the spectral space in which we define spectral neighborhoods and localize the SR mappings. In a second contribution, on discovering the best DNN degrades when images are rotated or blurred, we introduce those image manipulations in its training stage as part of a data augmentation process, which stabilizes its SR performance across those conditions (column (B) of Figure 2). Combined, we present experimental results which indicate (i) the pixel-based A++ generally outperforms the leading DNN across the concerned testing conditions (column (C) of Figure 2), (ii) A++ takes 1/20 the time to train, and (iii) A++ recovers spectra in 1/4 the time as the best DNN.

The rest of the paper is organized as follows. Section 2 reviews related works in SR. Section 3 presents our proposed new method. The experiment and results of the SR testing are reported in Section 4. In addition, in Section 5, we present a demonstration of using the concerned SR models for the scene relighting application. Section 6 concludes this paper.

## 2. Related Works

The earliest SR approaches seek 3-dimensional linear models of spectra. It is then shown that, if such a “3-D” linear model holds, the spectra can be exactly recovered from RGBs using a linear transform [28,38]. While a 3-D model can only cover limited variance of real-world spectra [39,40,41], simple statistical models such as regression [27,34,42] and Bayesian inference [26,43] are proposed, which supports higher- or full-dimensional spectral recovery. As the amount of available data has increased, recent methods are based on richer inference algorithms, including sparse coding [29,44], shallow networks [45,46,47] and deep neural networks (DNN) [29,30,35,48,49,50,51]. However, not all recent and early methods have been benchmarked on the same database so a fair overall comparison of the methods is not fully available. Yet, it would be fair to say that DNNs are accepted as the leading SR method.

Among the early methods, regression [27] is a popular approach due to its simple, fast, accurate and closed-form solution. The simplest “linear regression” [27] relates RGBs and their spectral estimates by a single linear transformation matrix. To introduce non-linearity, polynomial and root-polynomial regression [34,42] expand the RGBs into polynomial/root-polynomial terms, which are then mapped to spectra via a linear transform. Generally, “least-squares” regressions are considered, where the mean squared error (MSE) in the training set is minimized. However, because SRs are—at least latterly—more commonly evaluated using relative (percentage) errors [29,30,37,44], Lin and Finlayson [32] developed a “relative-error-least-squares” minimization approach for regressions, which further improves the performance of regression-based SR.

Unlike regression, where one single SR mapping is applied to all the input RGBs, sparse coding approaches [37,44,52] seek to determine multiple SR mappings that are used in different RGB neighborhoods. Recently Lin and Finalyson [53] proposed that instead of assigning local mappings in the RGB space, doing so in the spectral space can greatly improve the upper-bound performance of sparse coding—to the extent that it even far surpasses a top DNN [53]. Though, their argument employed the concept of an “oracle” that could always correctly locate the (practically unknown) ground-truth spectra among the spectral neighborhoods. In this paper, we seek to propose an approximated model that can probably help us approach the performance of this oracle solution.

Most of the recently proposed approaches to SR are based on DNN architectures—either convolutional neural networks (CNN) or generative adversarial networks (GAN)—where large image patches are standard inputs to the networks. In the recent NTIRE 2018 and 2020 Spectral Reconstruction Challenges [29,30], all top finalists are based on DNNs. In this paper, we consider two DNN models for comparison to our proposed method. First, “HSCNN-D” [50] is the 1st-place winner of the NTIRE 2018 challenge [29], which adopts a densely-connected structure. In addition, “AWAN” [35] is the winner of NTIRE 2020 challenge [30], which is based on the attention network structure. Despite those advances, most DNN benchmarks are carried out on ideally captured images (e.g., still images with well-adjusted exposures). The main ranking protocols of NTIRE competitions also do not account for performance under more difficult imaging conditions (that are still often encountered in the real world). Indeed, more comprehensive benchmarks show that DNNs are generally vulnerable to exposure change [33,34], out-of-scope scenes [30] and scenes without particular image contents [30,54]. In this paper, we will also show that the leading DNN is negatively and significantly affected by image rotation and blur.

## 3. A++ Pixel-Based Spectral Reconstruction

### 3.1. Preliminaries

Nowadays, most SR algorithms are trained on hyperspectral image datasets [44,45,55]. Here, and in most works, the RGB counterparts of spectra are formed by [1]:

(1) $$x_{c}=\int_{\Omega }\;s_{c}\left(\lambda \right)r\left(\lambda \right)d\lambda \;,$$

where r(λ) represents the physical radiance spectrum, $s_{c}\left(\lambda \right)$ is the *c*-th channel spectral sensitivities of the RGB sensors (c=R,G,B), and $x_{c}$ is the derived *c*-th channel RGB response. For RGB imaging, the effective range of wavelengths, Ω, is the visible range (roughly runs from 400 to 700 nanometers).

In practice, hyperspectral measurements are “discrete” at some sampled wavelengths. In this paper we consider Ω={400,410,⋯,700} meaning that the spectral samplings are every 10 nanometers from 400 to 700 nanometers, and so the hyperspectral images have 31 spectral channels. Hence, we write Equation (1) in a vectorized form:

(2) $$\underline{x}={\left[{\underline{s}}_{R},{\underline{s}}_{G},{\underline{s}}_{B}\right]}^{T}\underline{r}\;,$$

where $\underline{x}={\left[x_{R},x_{G},x_{B}\right]}^{T}$, and ${\underline{s}}_{R}$, ${\underline{s}}_{G}$, ${\underline{s}}_{B}$ and $\underline{r}$ are the 31-dimensional vectors of discretized $s_{R}\left(\lambda \right)$, $s_{G}\left(\lambda \right)$, $s_{B}\left(\lambda \right)$ and r(λ), respectively.

This RGB simulation methodology is important because it means that we have perfect ground truth (we know exactly the radiance spectrum associated with each RGB). All DNNs (the “leading” SR algorithms) estimate the spectra using an RGB and its surrounding pixels in an image patch. In contrast, pixel-based methods map RGBs to spectra without any knowledge of the image context.

### 3.2. Overview of A+ and A++

In sparse-coding-based SR, clustering techniques are used to help define neighborhoods in the RGB space [37,44,52]. In A+ [37], K-SVD clustering [56] is used to cluster the *spectral* data, and via the color formation formula (Equation (2)) we get *K* RGB clusters. Around the center of each cluster, a fixed number of *N* RGB neighbors are found in the training-set data, and together with their associated ground-truth spectra, we train a linear least-squares SR map that is associated with this cluster. In testing, we then find one out of the *K* clusters whose center is the closest to a given testing RGB, where the SR map associated with this cluster will be applied to the RGB to reconstruct spectrum.

As an extension of A+, in A++, we wish to cluster and localize mappings in the *output space* (spectral space). That is, we want to ensure that similar (neighboring) ground-truth spectra are recovered by the same mapping. Lin and Finlayson [53] directly manipulated the ground-truth spectra in the testing phase and force this to be true, as such to derive the upper-bound performance of this setup. While the result is appealing (the upper bound is far beyond the top DNNs’ performance), it is impossible to manipulate ground truths in the *actual* testing where they are unknown.

Our idea is to apply a “primary” SR algorithm to the training/testing RGBs, where these primary spectral estimates will be used to help us determine the spectral neighborhoods (instead of using the ground-truths). In essence, since the primary SR algorithm already estimates spectra, our sparse coding architecture can be viewed as a “post-refinement” process for the primary SR.

We summarize the training and testing (reconstruction) steps of our method in Table 1. We will dedicate the rest of Section 3 to providing details of these steps.

### 3.3. Primary SR Algorithm

The choice for our primary SR algorithm is not *a priori* fixed. For example, we may simply use the state-of-the-art DNN as the primary SR. Nevertheless, considering the balance between model complexity and performance (and also to ensure that our proposed method is a pixel-based mapping as per our research goal), we select the “6th-order polynomial regression with relative-error-least-squares minimization” (PR-RELS) [32] as our primary SR map. The PR-RELS was shown to perform less than 10% worse than a top-performing DNN [32].

In PR-RELS, we find a global linear transformation matrix, denoted as M, that maps the polynomial-expanded RGBs to spectra:

(3) $$M\varphi \left(\underline{x}\right)=\hat{\underline{r}}\approx \underline{r}\;,$$

where $\varphi (\underline{x})$ is a vector of polynomials of $x_{R}$, $x_{G}$ and $x_{B}$ (including the cross-terms) up to a given order [42], and $\hat{\underline{r}}$ denotes the primary spectral estimate. Assuming $\varphi (\underline{x})$ expands the RGB $\underline{x}$ into a *p*-term vector, M will then be a 31×p matrix (recall that 31 is the dimension of spectra $\underline{r}$).

The RELS minimization [32] solves M by minimizing:

(4) $$M=\underset{M}{\arg\;\min}\;\left(\sum_{i}||\frac{M\varphi \left({\underline{x}}_{i}\right)-{\underline{r}}_{i}}{{\underline{r}}_{i}}|{|}_{2}^{2}\right)\;,$$

where ${\underline{x}}_{i}$ and ${\underline{r}}_{i}$ are the *i*-th training ground-truth RGB and spectrum, and the division is component-wise to the vectors.

For the closed-form solution of Equation (4) and its regularization setting, readers are referred to [32]. In this paper, we assume PR-RELS has been pre-trained (with the same set of training data) prior to our sparse coding process.

### 3.4. Clustering Step

Using the PR-RELS map, we transform all training RGBs $\underline{x}$ to the primary estimates $\hat{\underline{r}}$. Then, we cluster those spectral estimates using the K-SVD clustering algorithm [56]. The cluster centers are selected into a dictionary:

(5) $$D=\left\{{\hat{\underline{r}}}^{1},\;{\hat{\underline{r}}}^{2},\;\cdots \;,\;{\hat{\underline{r}}}^{j},\;\cdots \;,\;{\hat{\underline{r}}}^{K}\right\}\;,$$

where the superscript ${}^{j}$ indexes the clusters, and a total of *K* clusters are determined.

Around each cluster center (i.e., member of D), we redefine its belonged cluster by finding the *N* closest primary estimates in the training set. These fixed-sized clusters may or may not overlap with other clusters (i.e., each training-set primary estimate can appear in one or more clusters). Taking the *j*-th cluster as an example, we write:

(6) $${\hat{R}}^{j}=\left[{\hat{\underline{r}}}_{1}^{j},\;{\hat{\underline{r}}}_{2}^{j},\;\cdots \;,\;{\hat{\underline{r}}}_{\ell }^{j},\;\cdots \;,\;{\hat{\underline{r}}}_{N}^{j}\right]\;,$$

where the columns of ${\hat{R}}^{j}$ are the *N* primary-estimate neighbors of ${\hat{\underline{r}}}^{j}$, and the subscript ${}_{\ell }$ indexes the neighbors.

Notice that here, and throughout the paper, the closeness is evaluated by the Euclidean distance between “normalized” vectors (i.e., all primary estimates are normalized into unit vectors upon calculating their distance with the cluster centers). This is because ${\hat{\underline{r}}}^{j}$ and all other members in D are normalized vectors as per the default setting of K-SVD.

There are two factors introduced in this clustering step that can greatly influence the performance of our method, which are *K*, the number of clusters, and *N*, the size of each cluster. The former decides how far the clusters are apart, while the latter adjusts how “overlapping” the adjacent clusters are. We will present the empirical search for both factors later in the experimental section (Section 4.4).

### 3.5. Local Linear SR Maps

#### 3.5.1. Training

Clearly, we can trace back to the training RGB and ground-truth spectrum associated with each primary estimate in the columns of ${\hat{R}}^{j}$. We then arrange those RGBs and spectra into corresponding columns of $X^{j}$ and $R^{j}$. Then, a local linear map can be formulated as:

(7) $$M^{j}X^{j}\approx R^{j}\;,$$

where $M^{j}$ is a 31×3 local linear regression SR associated with the cluster *j*.

Same as in A+ [37], we solve $M^{j}$ using the closed-form regularized least-squares minimization [27,57]:

(8) $$\begin{array}{cc} M^{j} & =\underset{M^{j}}{\arg\;\min}\;\left(||M^{j}X^{j}-R^{j}|{|}_{F}^{2}+\gamma ||M^{j}|{|}_{F}^{2}\right)\\ \; & =R^{j}{\left[X^{j}\right]}^{T}{\left(X^{j}{\left[X^{j}\right]}^{T}+\gamma I\right)}^{-1}\;, \end{array}$$

where $\left|\right|\cdot {\left|\right|}_{F}$ denotes the Frobenius norm, I is the 3 × 3 identity matrix, and ${}^{T}$ denotes matrix transpose.

Here, the γ parameter (i.e., the regularization parameter) bounds the norm of $M^{j}$ in the minimization. Determining the proper γ value is often empirical. In this paper we use the cross validation approach [58], where a range of different γ values are tried to recover spectra from the RGBs in a separated “validation dataset” and in the same (here, the *j*-th) cluster, and the one that minimizes the mean reconstruction error on this separate dataset is selected. Our search range for γ is between $\left[10^{-20},10^{20}\right]$ (although we note that for linear regressions choosing a fixed small γ almost always delivers close to optimal performance).

#### 3.5.2. Testing

Since there are *K* clusters (whose centers are recorded in D), we have *K* linear mappings in the form of Equation (8) (each for the cluster with the corresponding label). To determine which mapping to use for each testing RGB, again denoted as $\underline{x}$, we first transform it into a primary estimate, $\hat{\underline{r}}$, using PR-RELS (Equation (3)), and then find which cluster center in D is the closest to $\hat{\underline{r}}$. The linear mapping associated with the closest cluster center will then be applied to $\underline{x}$ to deliver the final SR output.

We point out that, although in training, the same RGB can be included in multiple clusters and used to train separate local maps (because clusters are allowed to overlap), in testing each testing RGB will only associate with one cluster—only the closest cluster center, or say the *best cluster*, is selected.

## 4. Experiments

In this section, we will benchmark our method against two of the top-performing DNNs: HSCNN-D [29,50] and AWAN [30,35], as well as the pixel-based A+ sparse coding [37] and PR-RELS regression methods [32]. Our A++ method combines aspects of the latter two methods.

According to the recommendations in respective citations, we set the depth of HSCNN-D to 240 (i.e., equivalent to 58 dense blocks) [50], and our AWAN implementation uses 8 dual residual attention blocks (DRAB) with 200 output channels set for their patch-level second-order non-local (PSNL) module [35].

All models will be tested on the original, rotated and blurred testing images. We will also introduce how we tune the hyperparameters of our A++ sparse coding architecture and our data augmentation attempt for AWAN.

The implementation codes are submitted as the supplementary materials.

### 4.1. Dataset

We use the ICVL benchmarking hyperspectral dataset [44], which was the basis for the NTIRE 2018 SR challenge [29]. ICVL comprises 200 scenes captured both indoors and outdoors. The size of each image is 1300 × 1392, and at each pixel, the spectral signal is recorded in 31 channels, referring to the discrete spectral measurements from 400 to 700 nanometers (nm) with 10-nm intervals.

The corresponding RGB images are derived from the hyperspectral images using Equation (2), with CIE 1964 color matching functions [59] as the spectral sensitivities.

This dataset setting aligns with the “clean track” of NTIRE 2018 and 2020 SR challenges [29,30].

### 4.2. Training, Validation and Testing

From the dataset, we randomly separate the hyperspectral/RGB image pairs into 100 pairs for training, 50 pairs for testing, and 50 pairs for *model validation* (i.e., for determining regularization parameter γ in Equation (8), or for determining the ending epochs of DNN trainings).

To speed up the training process of A+ and A++, we train both models with only a fraction of the training data (this is possible because sparse coding methods, compared to DNNs, need fewer data to train). We randomly select 3000 pixels per training scene for the clustering training (Equation (5)), and 30,000 pixels per scene for determining the fixed-sized clusters (Equation (6)).

As for the DNNs (AWAN and HSCNN-D), we use the complete images in training and validation. We stop iterating HSCNN-D until the training loss does not decay anymore, while for AWAN, we set the maximum epoch at 25.

The reference information of the number of model parameters (indicating the model complexity), consumed training time and testing (reconstruction) time is given in Table 2. Our equipment includes Intel${}^{\text{®}}$ Core${}^{\mathrm{TM}}$ i7-9700 CPU and NVIDIA${}^{\text{®}}$ GeForce${}^{\text{®}}$ RTX 2080 SUPER${}^{\mathrm{TM}}$ GPU. The GPU is only used to train the DNNs. All testing, as well as the training of pixel-based methods, only involve the CPU.

Evidently, similar to the pixel-based A+ and PR-RELS, our A++ method uses much fewer model parameters (about 8% as much as AWAN uses), which leads to much faster training and reconstruction.

### 4.3. Evaluation Setup

In the robustness testing, we create a rotated test set which consists of the 50 original testing images rotating by 90 degrees clockwise. As for the blurred test set, we apply 2-D Gaussian filters to the original testing images, with two different standard deviation (σ) settings: σ=10 and σ=20 (unit: pixels). Moreover, when applying the Gaussian filters at border pixels, the outer margins of the images are reflected with respect to the edges (i.e., the “half-sample symmetric” approach [60]).

The metric used for testing the SR efficacy is the often-used Mean Relative Absolute Error (MRAE) [29,30]:

(9) $$\mathrm{MRAE}\;\left(\%\right)=\frac{1}{31}||\frac{\hat{\underline{r}}-\underline{r}}{\underline{r}}|{|}_{1}\times 100\;,$$

where $\hat{\underline{r}}$ and $\underline{r}$ denote the reconstructed and ground-truth spectrum at a pixel, the division is component-wise to the vectors, and $\left|\right|\cdot {\left|\right|}_{1}$ refers to the $\ell_{1}$ (Taxicab) norm. The $\frac{1}{31}$ factor signifies that MRAE measures the mean error over the 31 spectral channels. In this paper, we present MRAE in *percentages* since in MRAE, the error is calculated with respect to the ground truth, which is a percentage error by nature.

We use MRAE because it is the standard protocol for evaluating and ranking the modern DNN-based SR approaches [29,30]. Many top DNNs also directly optimize for this metric, including the HSCNN-D and AWAN models [35,50]. For a more in-depth explanation on why MRAE is more suitable than the common Root-Mean-Squared Error (RMSE) for SR evaluation, we point the readers to [32].

### 4.4. Tuning Our A++ Sparse Coding Architecture

As mentioned in Section 3.4, there are 2 hyperparameters in A++ that could potentially influence the performance: the number of clusters (*K* in Equation (5)) and the size of each cluster (*N* in Equation (6)).

The original A+ model [37] uses (K,N)=(1024,8192), and yet this might not be the best setting for our new setup. So, we are to re-determine both factors.

We start with fixing N=8192 and search for the best *K* setting. We experiment on original testing images (no rotation, no blur) and calculate the mean per-image-mean-MRAE over the test set. The result is shown in the upper Table 3, which suggests that K=8192 is the best setting. Then, we, in turn, fix *K* at this value and search for *N*. It is shown in the lower Table 3 that N=1024 returns the lowest error. Therefore, we use (K,N)=(8192,1024) for our A++ implementation.

### 4.5. DNN Data Augmentation

In this paper, we add a data augmentation step to the AWAN DNN model [35], so the networks can account for rotation and blur. We do not also data-augment HSCNN-D [50] because, as will be shown later in the result section, HSCNN-D is more stable against both conditions.

Although we only test the models with one condition at a time (either rotation or blur), we shall still ensure that the data-augmented AWAN can adapt to more extensive changes. For each training/validation image inputted to the network, we are to randomly decide both of the following:

- one out of four image orientations including the original, 90 degrees, 180 degrees and 270 degrees clockwise, and
- a σ factor for the Gaussian filter, drawn from the uniform distribution between [0,20].

Both conditions are applied consecutively to the input image (the order does not matter). Then, the processed image will be—in replacement for the original image—used to train the AWAN network.

Notice that for training the data-augmented model, we increase the polynomial decay power of their adaptive learning rate from the original 1.5 to 15, which ensures better training-loss convergence.

**Figure 3:**
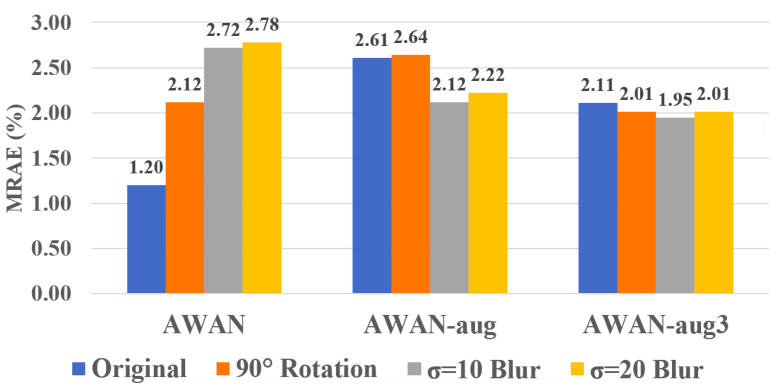
The effectiveness of our data augmentation setups for AWAN. The AWAN-aug result refers to augmenting input images with one random condition (a combined condition of rotation and blur), while AWAN-aug3 augments 3 random conditions per image. The results are shown in mean per-image-mean-MRAE.

As shown in the left-most result in Figure 3, on average, the non data-augmented AWAN works well on the original image, but has almost twice as much error for rotated images and performs even worse on blurred images. With data augmentation (the middle “AWAN-aug” result), we see that the model delivers a more stable performance across different conditions but at a worse overall performance level.

Considering that perhaps adopting only one random condition per image is not enough for the network to learn the variation, we try augmenting the network with 3 random conditions per image (the right-most result in Figure 3). Evidently, this “AWAN-aug3” setting provides even better stability and overall performance across all testing conditions. In the following section, we will include AWAN-aug3 in the benchmark with other compared methods.

### 4.6. Results

We present the mean and 99-percentile (i.e., the “worst-case”) performance of all considered models and imaging conditions in Table 4. For each image, we first calculate the mean and 99-percentile MRAE across its pixels, i.e., the “per-image-mean” and “per-image-99-percentile” MRAE. Next, we calculate the mean of these per-image statistics across the testing image set, provide the final presented mean and worst-case statistics.

In terms of the models’ mean performance, we see the best-performing model under the original testing condition (headlined “Orig”) is the DNN-based AWAN. In fact, it performs considerably better than all the rest of the models. However, it also suffers the most when the 90${}^{\circ }$-rotation (“Rot90”) and blur conditions (“Blur10” and “Blur20”) are introduced. While the HSCNN-D and AWAN-aug3 provide much more stable performance across the testing conditions, they do not perform as well as our proposed method.

This result shows a key advantage of pixel-based approaches that, perforce, they are independent of where the pixel is positioned in an image, and so the image orientation does not change the SR outcomes. Equally, assuming the pixel-based methods are well regularized (not overly fit to the training data), small perturbations in the RGB value should result in small perturbations in the recovered spectrum [32], which suggests that pixel-based SRs are resilient in the face of image blurring. In contrast, the blurring condition prevents the patch-based DNNs from inferring using the high-frequency content in the image.

Next, the worst-case results (right-hand-side Table 4). We see that under the original testing condition, the DNNs generally have better worst-case performance compared to the pixel-based methods. However, their advantage does not hold when the rotation and blur conditions are introduced, where the pixel-based PR-RELS takes the lead.

**Figure 4:**
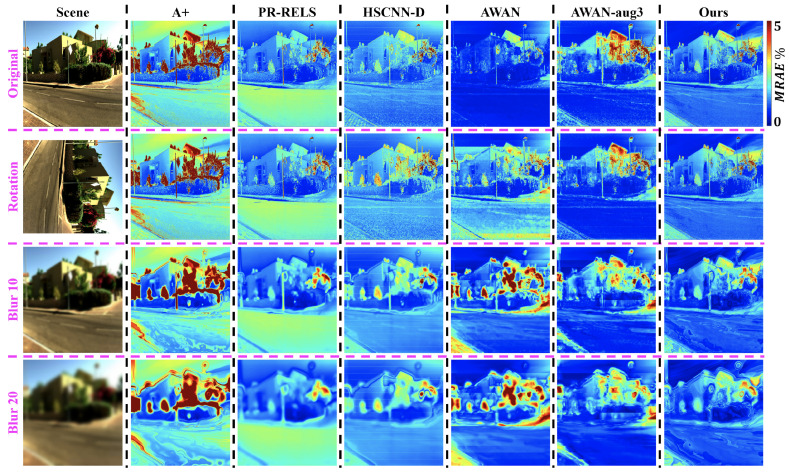
An example visualized hyperspectral image reconstruction performance by all compared methods. One scene from the ICVL database [44] shown in the left-most column is tested under the original (**top row**), rotation (**middle row**), and two Gaussian blur conditions (**bottom 2 rows**). The error maps for the rotation condition are rotated back to an upright orientation to ease the comparison.

Example hyperspectral image reconstruction results are visualized in Figure 4. Clearly, A++ significantly improves from the pixel-based baselines i.e., using A+ and PR-RELS individually, while retaining their robustness against image rotation and blur. On the other hand, while the effectiveness of our data augmentation setup on AWAN-aug3 is evident, its overall performance is still inferior to our proposed pixel-based A++ method.

**Figure 5:**
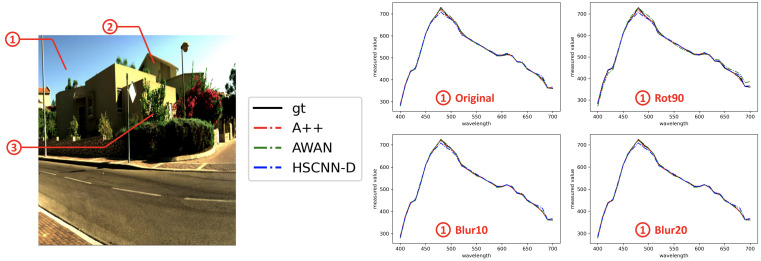
Visualization of selected ground-truth and recovered spectra (continued in Figure 6). **Left:** 3 pixels specified in an example scene. **Middle:** Legend for the spectral plots—in all plots in Figure 5 and Figure 6, ground-truth (gt) is shown in black, A++ in red, AWAN in green, and HSCNN-D in blue. **Right:** The recovery of spectra in the “sky” region (i.e., region ➀ in the example scene) under the Original, Rot90, Blur10 and Blur20 imaging conditions.

**Figure 6:**
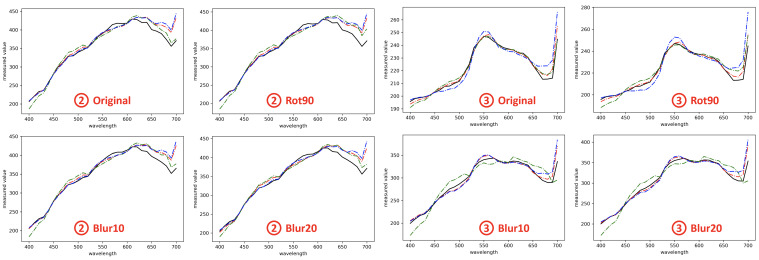
Visualization of the ground-truth and recovered spectra in region ➁ and ➂ in the example scene in Figure 5. The legend for the different colored curves is the same as in Figure 5: ground-truth (gt) is shown in black, A++ in red, AWAN in green, and HSCNN-D in blue. Respectively, region ➁ refers to the “building” and region ➂ the “plants”.

In Figure 5 and Figure 6, we visualize the spectral recovery results of A++, AWAN and HSCNN-R in comparison to the ground-truth at three selected pixels of an example scene—the *sky*, *building*, and *plants*. The results under the original, rotation and blurring are also shown separately. It is clear that the rotation and blurring effects cause AWAN to deteriorate, and in the third example (➂ on the right of Figure 6), we see the degradation of AWAN under blurring effect can be very significant. It is also shown that A++ performs on par with HSCNN-D in example ➀ and ➁, and better in example ➂.

#### Characteristic Vector Analysis Test

Another way of looking at the feasibility of a reconstructed dataset is by conducting Characteristic Vector Analysis (CVA) [61] and comparing its outcome with the ground-truth’s (A well-known variant of CVA is the Principal Component Analysis (PCA). In PCA, we conduct CVA while the vector of the mean values of all feature dimensions is subtracted from all data points [61]). In CVA, we find characteristic vectors in the feature space that—in descending order—maximally explain the variance in data and are orthogonal to all previous characteristic vectors. Practically, CVA is often used to reduce the dimensionality of a dataset by selecting only the top few characteristic vectors and representing all data points as linear combinations of these components. On the other hand, given two spectral datasets—one ground-truth and one reconstructed—by comparing their top characteristic vectors and the *eigenvalues* of these vectors (aka the “explained variance” by each characteristic vector), we can conclude how alike these two datasets are.

**Figure 7:**
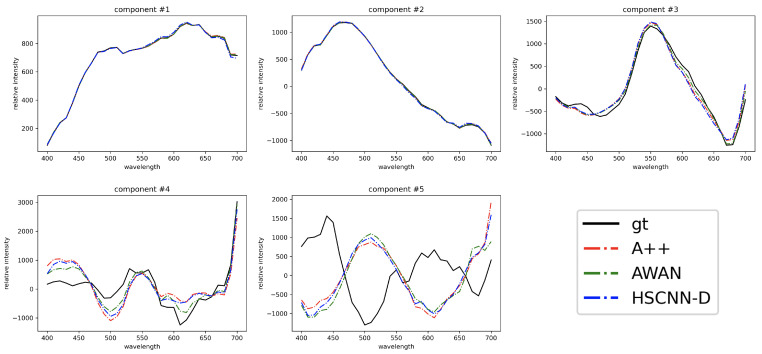
The top 5 Characteristic Vector Analysis (CVA) characteristic vectors of the ground-truth (gt; black curve), A++-recovered (red), AWAN-recovered (green) and HSCNN-D-recovered spectra (blue) in the testing image set. All recovered spectra are from original testing images without rotation or blurring.

In Figure 7, we compare the top 5 CVA characteristic vectors of the recovered spectra (by HSCNN-D, AWAN and A++) and ground-truth spectra in the testing image set. Evidently, the first 3 characteristic vectors of all three reconstructed spectral datasets are very similar to the ground-truth dataset. Clear discrepancies start to appear in the 4th component, and the 5th component of all algorithms are drastically different from the ground-truth’s. Nevertheless, the similarity of the 4th and 5th characteristic vectors among reconstruction algorithms remains high. This means that our proposed pixel-based A++ algorithm can recover a spectral dataset similar to the datasets recovered by the DNN-based AWAN and HSCNN-D. We can also see that as shown in Table 5, the respectively explained variances of the top 5 characteristic vectors of all algorithms are broadly in the same order of magnitude as the ground-truth’s.

### 4.7. Discussion and Limitations

While AWAN does not work well under more general realistic conditions, we do notice that it provides leading performance on the original testing images. In other words, if for some domain-specific tasks, the image orientation can be fixed and the image blur is the same as in testing (e.g., viewing fixed objects), then AWAN is a good candidate.

One might also argue that we could calibrate the image orientation and deblur the images prior to SR, or augment more data with perhaps a more complex or deeper network structure, and as such, it is still possible for AWAN to surpass A++ for those realistic conditions. However, all of these additional processes effectively add more computational complexity to what appears to be already complex (making AWAN even less approachable in practice). In contrast, A++ is a much simpler and equally effective SR solution that has lower hardware requirements than the DNNs—which is an import factor to consider if we would like to implement the algorithm on, for example, drones, embedded systems, etc.

We want also to point out that, although as per our research interest (to see whether patch information is needed for top-performing SR), we design A++ to be a pixel-based method, a pixel-based mapping fundamentally cannot distinguish materials of the same RGB (since the same RGB will always map to the same spectral estimate). This limitation goes against the premise that hyperspectral imaging can distinguish materials that are not distinguishable by an RGB camera. Hence, for applications where this ability is crucial, A++ and all other pixel-based methods may not be competent. However, they still serve as a baseline to see if the patch-based DNNs indeed perform better in this regard.

Even though we are presenting a pixel-based algorithm, what we want to show here is that currently, the best DNNs do not perform better than the best pixel-based methods, and this calls into doubt the extent to which these algorithms can map the same RGB to different spectra depending on context. This does not mean we do not recognize the DNNs’ premise—that materials and/or objects are identified deep in the network—is good. Unfortunately, that premise is not delivered upon in the architectures that are currently used. We believe our development of A++ will encourage future research on simpler spectral reconstruction techniques as well as more mindful and efficient designs for DNN-based solutions.

## 5. Demonstration: Spectral Reconstruction for Scene Relighting

**Figure 8:**
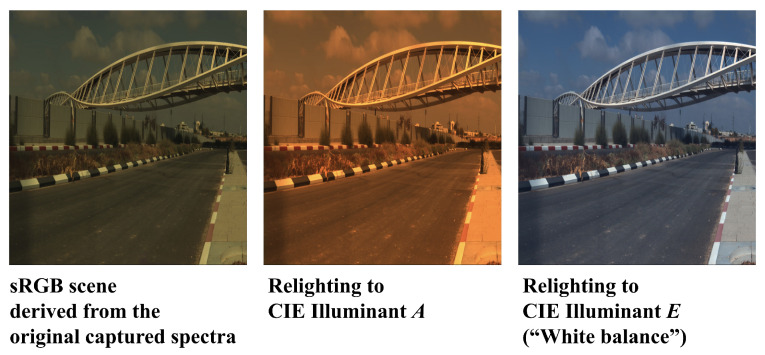
The original (**left**) and relighted scenes (**middle** and **right**) shown in sRGB colors.

Scene relighting refers to changing the light source of the scene as a computational process (instead of physically changing the light source), which predicts how the RGB colors would appear under the target illumination (Figure 8).

In most color imaging applications where the illumination of the scene is manipulated (e.g., most significantly the color constancy or white balancing application), an “RGB diagonal model” is assumed [62], which suggests that the relighted RGBs, ${\underline{x}}^{\prime }$, are related to the original RGBs, $\underline{x}$, by:

(10) $${\underline{x}}^{\prime }\approx diag\left(\frac{\;{\underline{l}}_{c}^{\prime }\;}{\;{\underline{l}}_{c}\;}\right)\underline{x}\;,$$

where ${\underline{l}}_{c}^{\prime }$ and ${\underline{l}}_{c}$ are, respectively, the RGB colors of the target and original light sources (a.k.a. their “white points”). Here, the division is component-wise, and the diag() function turns a vector into a diagonal matrix (the vector makes up the diagonal entries of the matrix with zeros elsewhere).

In this demonstration, we evaluate how scene relighting via SR (which will be introduced later) works in comparison to the traditional RGB diagonal method (Equation (10)).

### 5.1. “Ground-Truth” Scene Relighting

Theoretically, the RGB diagonal model is only exact when $\underline{x}={\underline{l}}_{c}$ (in which case ${\underline{x}}^{\prime }={\underline{l}}_{c}^{\prime }$ is the correct answer), and yet for all other RGBs this model is only an assumption-based approximation (and thus the ≈ symbol in Equation (10)).

With the help of hyperspectral imaging, we can derive physically accurate scene relighting for all RGBs. Returning to Equation (2), where we described that RGB $\underline{x}$ is formed by $\underline{x}={\left[{\underline{s}}_{R},{\underline{s}}_{G},{\underline{s}}_{B}\right]}^{T}\underline{r}$ where $\underline{r}$ is the measured radiance spectrum and $\left[{\underline{s}}_{R},{\underline{s}}_{G},{\underline{s}}_{B}\right]$ is the RGB camera’s spectral sensitivities. In fact, the measured $\underline{r}$ can be further separated into two independent components: the illumination spectrum $\underline{l}$ (intrinsic to the light source) and the object’s surface reflectance $\underline{\rho }$ (intrinsic to the object surface). Assuming the world is strictly composed of flat and matte surfaces, we write [1,63]:

(11) $$\underline{r}=diag\left(\underline{l}\right)\underline{\rho }\;.$$

Given this simple physical model, we can formulate ground-truth scene relighting as:

(12) $$\begin{array}{cc} \; & {\underline{r}}^{\prime }=diag\left({\underline{l}}^{\prime }\right)\underline{\rho }=diag\left(\frac{\;{\underline{l}}^{\prime }\;}{\;\underline{l}\;}\right)\underline{r}\\ ⟹\; & {\underline{x}}^{\prime }={\left[{\underline{s}}_{R},{\underline{s}}_{G},{\underline{s}}_{B}\right]}^{T}{\underline{r}}^{\prime }\;, \end{array}$$

where ${\underline{l}}^{\prime }$ is the given target illumination spectrum we wish to relight the scene to, ${\underline{r}}^{\prime }$ is the relighted radiance spectrum, and ${\underline{x}}^{\prime }$ is the *exact* relighted RGB (i.e., the “ground-truth”).

### 5.2. Experiment: SR Relighting vs. RGB Diagonal Model Relighting

Given the RGB data, instead of adopting the RGB diagonal model assumption in Equation (10), SR algorithms estimate the radiance spectrum $\underline{r}$ from the RGBs, which enables us to use the physical model (Equation (12)) for scene relighting. While the efficacy of the RGB diagonal model is subject to how well the assumption holds, the SR relighting approach is influenced by the SR accuracy.

We examine the efficacy of SR relighting delivered by all considered SR algorithms and under all concerned realistic imaging conditions (rotation and blur). The performance of the traditional RGB diagonal model is also presented as a baseline. The ground-truth relighted RGB images are derived from the ground-truth hyperspectral images using the physical model in Equation (12).

Specifically, we relight all test scenes (defined in Section 4.2) to the standard CIE Illuminant *A* and Illuminant *E* [64], where the former represents a tungsten-filament light source with a color temperature around 2856 K, and the latter is the quintessential “white spectrum” (that is, a hypothetical spectrum with a constant intensity across all wavelengths). We study relighting to a white spectrum because it resembles the white balancing process, which normally adopts the RGB diagonal model.

We also need to know the original illumination spectrum $\underline{l}$ (or for the RGB diagonal model, the original illumination color ${\underline{l}}_{c}$) to operate scene relighting. Therefore, we estimate $\underline{l}$ using the “white patch” approach [65]. In particular, we set $\underline{l}$ as the hand-crafted brightest achromatic spectrum in each hyperspectral image, where *brightness* is defined as the $\ell_{2}$ norm of the spectrum (the illumination color ${\underline{l}}_{c}$ can then be derived from the hand-crafted $\underline{l}$ using RGB simulation: ${\underline{l}}_{c}={\left[{\underline{s}}_{R},{\underline{s}}_{G},{\underline{s}}_{B}\right]}^{T}\underline{l}$).

#### 5.2.1. Evaluation Metric

We wish to evaluate the scene relighting color error at each pixel using the CIE 2000 color difference ($\Delta E_{00}$) [66]. To calculate $\Delta E_{00}$, we are to transform both the ground truth and the compared estimated relighted RGBs to CIELAB colors [67]. Given that our RGBs are, in effect, the CIEXYZ tristimulus values (because we use the CIE color matching functions to simulate the RGBs; see Section 4.1), there is a direct transformation from CIEXYZ to CIELAB given the target relighting illumination color ${\underline{l}}_{c}^{\prime }$ [68].

We choose $\Delta E_{00}$ as our color error metric because it provides homogeneous measurements of color differences. More specifically, a $\Delta E_{00}=1$ indicates the “just noticeable difference” between two colors (below which a standard human observer cannot tell their differences). Implementing $\Delta E_{00}$ is rather complicated. Interested readers are pointed to [66] for more details.

#### 5.2.2. Results

The CIE Illumination *A* and *E* relighting results are presented in Table 6 and Table 7, respectively. We present the mean and worst-case (99-percentile) performance of all considered SR models and imaging conditions. Both statistics are calculated per image and then averaged over the test set.

First, we observe that all SR methods provide better scene relighting performance compared to the traditional RGB diagonal model, for both the mean and worst-case results. Specifically, the mean relighting accuracy via SR is generally very good ($\Delta E_{00}<1$ which is less than the human’s perceivable difference). Arguably, here, bounding the worst-case performance might be more important. Indeed, we see that for all methods the worst-case $\Delta E_{00}>1$, which might inflict perceivable color-shift defects in the resulting relighted images.

Next, although in our experiment the most accurate and robust SR methods (A++ and PR-RELS) also suggest the best results in scene relighting, in general, better spectral accuracy does not always imply better relighting performance. For instance, the DNN-based HSCNN-D provides much more accurate SR than the pixel-based A+ sparse coding (Table 4), but does not show many advantages over A+ in CIE Illuminant *A* relighting (Table 6). For another example, we see that even though AWAN-aug3 provides better spectral accuracy than AWAN on rotated and blurred images, its advantage does not reflect on the scene-relighting application, specifically if we look at its worst-case performance. However, we note that not performing well on scene relighting also does not necessarily mean an SR algorithm would fail in other applications (in which case the spectral accuracy may account for more of the performance).

Finally, we see that under the original testing condition, the DNN-based AWAN method provides the best relighting results overall, while for the robustness tests concerning image rotation and blur, the pixel-based PR-RELS and our proposed A++ methods come to the fore.

**Figure 9:**
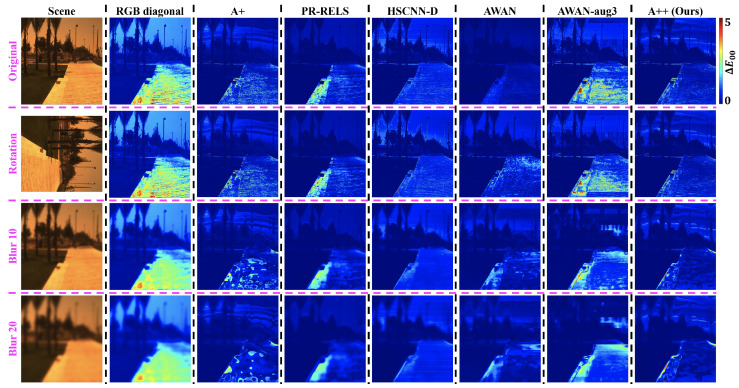
CIE Illuminant *A* scene relighting error heat maps in $\Delta E_{00}$. The ground-truth relighted scene is shown in sRGB in the leftmost column. From the top to the bottom row, the tested imaging condition is in turn the original, rotation, and two Gaussian blur conditions.

**Figure 10:**
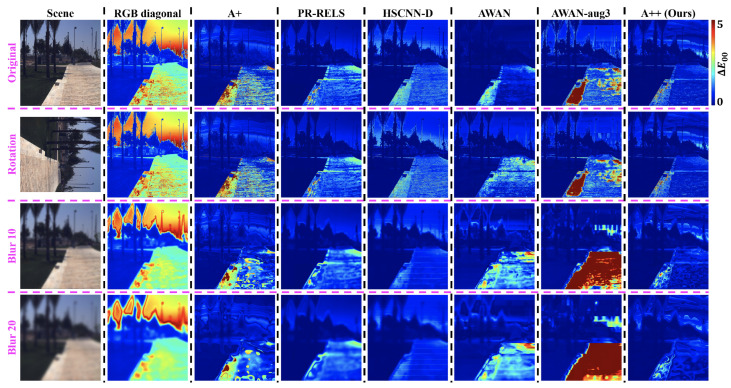
CIE Illuminant *E* scene relighting error heat maps in $\Delta E_{00}$. The ground-truth relighted scene is shown in sRGB in the leftmost column. From the top to the bottom row, the tested imaging condition is in turn the original, rotation, and two Gaussian blur conditions.

Visualized $\Delta E_{00}$ error maps for CIE Illuminant *A* and *E* relighting are respectively presented in Figure 9 and Figure 10. Evidently, relighting via SR algorithms generally provide much better color accuracy than the traditional RGB diagonal process, and our proposed SR method A++ provides the best accuracy and robustness in scene relighting overall.

## 6. Conclusions

The spectral reconstruction (SR) problem studies the recovery of light’s spectral signals from the RGB camera responses, which is regarded as a physics-based computer vision problem. In this work, we challenged ourselves to surpass the leading deep neural networks (DNN) in SR using only a pixel-based mapping model. We developed a new sparse coding architecture, called “A++”, where an RGB is mapped to the spectrum, firstly by a polynomial regression SR, and secondly by a linear SR map depending on the location of its first estimation in the spectral space. We show that this A++ method—despite being much simpler than the leading DNNs—delivers leading spectral accuracy across a range of realistic imaging conditions, including image rotation and blur. While we also addressed the discovered leading DNN’s robustness issue via a data augmentation process, our A++ method still delivers consistently better performance than the augmented DNN. A practical study on applying SR to the scene relighting application also shows the superior performance of A++ compared to the DNNs. Combined, we see that not only does our pixel-based A++ deliver SR of leading performance and robustness, but its lack of heavy DNN structures also ensures much faster training and real-time processing.

## Figures and Tables

**Table 1:** A summary of the training and testing (reconstruction) process of A++.

Training Steps		Testing (Reconstruction) Steps	
1.	Obtain primary SR estimates of all training RGBs	1.	Obtain the primary SR estimate of each testing RGB
2.	Run K-SVD clustering on the primary estimates	2.	Find the closest cluster center of this primary estimate
3.	For each cluster, find *N* RGBs in the training set whose primary estimates are closest to the cluster center	3.	Get the trained local linear SR map associated with this cluster
4.	Train a linear SR map associated with this cluster using the found *N* RGBs and their ground-truth spectra	4.	Apply this map to the testing RGB to reconstruct its spectrum

**Table 2:** The reference number of model parameters, training time and testing (reconstruction) time.

Method	Number of	Training	Testing Time
	Parameters	Time	(per Image)
HSCNN-D	9.3 × $10^{6}$	2.7 days	13.3 min
AWAN	1.7 × $10^{7}$	2.8 days	20.1 min
A+	9.5 × $10^{4}$	26.9 min	17.8 s
PR-RELS	2.6 × $10^{3}$	15.1 min	6.5 s
A++ (Ours)	7.6 × $10^{5}$	3.4 h	5.4 min

**Table 3:** The mean per-image-mean-MRAE performance in relation to the number of clusters (*K*) and the size of each cluster (*N*) used in our A++ method. The best result for each factor (while the other factor is fixed) is shown in bold font.

*K*	1024	2048	4096	**8192**	10,240
*N* (fixed)	——————8192——————				
MRAE (%)	1.88	1.82	1.78	**1.76**	1.78
*K* (fixed)	——————8192——————				
*N*	512	**1024**	2048	4096	8192
MRAE (%)	1.70	**1.69**	1.70	1.72	1.76

**Table 4:** The mean and 99-percentile hyperspectral image reconstruction error in MRAE, testing with the original test-set images (**Orig**), the 90${}^{\circ }$-rotated test-set images (**Rot90**), and the Gaussian blurred images with σ=10 (**Blur10**) and σ=20 (**Blur20**). The best methods in each experiment are in bold font, and the second bests are underlined.

Approach	Method	Mean per-Image-Mean MRAE (%)				Mean per-Image-99-pt. MRAE (%)			
		Orig	Rot90	Blur10	Blur20	Orig	Rot90	Blur10	Blur20
DNN	HSCNN-D	1.71	1.91	1.70	1.70	7.18	7.76	6.97	6.54
	AWAN	**1.20**	2.12	2.72	2.78	**6.15**	8.08	10.75	10.34
	AWAN-aug3	2.11	2.01	1.95	2.01	9.60	9.17	9.51	9.20
Pixel-based	A+	3.81	3.81	3.70	3.71	15.52	15.52	14.36	13.47
	PR-RELS	1.86	1.86	1.70	1.70	7.56	**7.56**	**6.80**	**6.32**
	A++ (Ours)	1.69	**1.69**	**1.53**	**1.54**	8.11	8.11	7.30	6.85

**Table 5:** The variance of the testing spectra recovered by HSCNN-D, AWAN and A++, and ground-truths explained by their respective top 5 (#1 to #5) CVA characteristic vectors.

Method	Explained Variance (CVA Eigenvalue)				
	#1	#2	#3	#4	#5
HSCNN-D	$2.98\times 10^{-1}$	$1.31\times 10^{-2}$	$1.34\times 10^{-3}$	$2.64\times 10^{-4}$	$1.49\times 10^{-4}$
AWAN	$3.00\times 10^{-1}$	$1.36\times 10^{-2}$	$1.49\times 10^{-3}$	$3.21\times 10^{-4}$	$2.24\times 10^{-4}$
A++ (Ours)	$3.01\times 10^{-1}$	$1.35\times 10^{-2}$	$1.36\times 10^{-3}$	$2.44\times 10^{-4}$	$1.38\times 10^{-4}$
Ground-Truth	$2.99\times 10^{-1}$	$1.36\times 10^{-2}$	$1.73\times 10^{-3}$	$3.78\times 10^{-4}$	$2.86\times 10^{-4}$

**Table 6:** The CIE Illuminant *A* relighting results. The mean results of per-image mean and 99-percentile $\Delta E_{00}$ are presented. The best methods in each experiment are marked in bold font, and the second bests are underlined.

Approach	Method	Relighting to CIE Illuminant *A*							
		Mean per-Image-Mean $\Delta E_{00}$				Mean per-Image-99-pt. $\Delta E_{00}$			
		Orig	Rot	Blur10	Blur20	Orig	Rot	Blur10	Blur20
Baseline	RGB Diagonal	0.83	0.83	0.82	0.81	2.63	2.63	2.30	2.20
DNN-basedSR	HSCNN-D	0.30	0.30	0.24	0.24	2.28	2.36	1.82	1.71
	AWAN	**0.10**	0.20	0.32	0.32	**1.39**	1.90	1.91	1.81
	AWAN-aug3	0.23	0.23	0.15	0.15	2.33	2.35	1.95	1.84
Pixel-basedSR	A+	0.30	0.30	0.26	0.26	2.41	2.41	2.08	1.93
	PR-RELS	0.19	0.19	0.16	0.16	1.97	1.97	1.79	1.66
	A++ (Ours)	0.15	**0.15**	**0.13**	**0.13**	1.84	**1.84**	**1.70**	**1.62**

**Table 7:** The CIE Illuminant *E* relighting (the “white balancing”) results. The mean results of per-image mean and 99-percentile $\Delta E_{00}$ are presented. The best methods in each experiment are marked in bold font, and the second bests are underlined.

Approach	Method	Relighting to CIE Illuminant *E*							
		Mean per-image-mean $\Delta E_{00}$				Mean per-image-99-pt. $\Delta E_{00}$			
		Orig	Rot	Blur10	Blur20	Orig	Rot	Blur10	Blur20
Baseline	RGB diagonal	1.35	1.35	1.35	1.35	3.39	3.39	3.24	3.18
DNN-basedSR	HSCNN-D	0.33	0.34	0.27	0.26	2.58	2.74	2.03	1.92
	AWAN	**0.12**	0.21	0.24	0.24	**1.64**	2.11	2.21	2.14
	AWAN-aug3	0.27	0.27	0.23	0.24	3.00	2.97	2.92	2.85
Pixel-basedSR	A+	0.40	0.40	0.36	0.35	3.17	3.17	2.74	2.59
	PR-RELS	0.17	0.17	0.16	0.15	1.87	**1.87**	**1.78**	**1.68**
	A++ (Ours)	0.16	**0.16**	**0.13**	**0.13**	1.99	1.99	1.81	1.73

## Data Availability

Publicly available datasets were analyzed in this study. This data can be found here: http://icvl.cs.bgu.ac.il/hyperspectral/ (accessed on 12 April 2023).

## Supplementary Materials

The code of the methods introduced in this paper is available at https://github.com/EthanLinYitun/A_Plus_Plus_Spectral_Reconstruction (accessed on 12 April 2023).
